# Effect of Enzyme Treatment on Physicochemical and Phytochemical Properties of Guava Puree

**DOI:** 10.1002/fsn3.70939

**Published:** 2025-11-10

**Authors:** M. L. Plaza, M. M. Ramírez‐Rodrigues, M. O. Balaban, M. R. Marshall

**Affiliations:** ^1^ Department of Agroenvironmental Science, Food Science and Technology Program University of Puerto Rico at Mayaguez Mayaguez Puerto Rico; ^2^ Department of Food Science and Nutrition California Polytechnic State University San Luis Obispo California USA; ^3^ Department of Chemical and Materials Engineering University of Auckland Auckland New Zealand; ^4^ Food Science and Human Nutrition Dept University of Florida, Food and Environmental Toxicology Lab Gainesville Florida USA

**Keywords:** enzyme treated, guava juice, physicochemical properties, *Psidium guajava*

## Abstract

Guava ( *Psidium guajava* L.) is underutilized due to the presence of excessive suspended solids that hinder product clarity and consumer acceptability. In this study, the enzymatic treatment conditions (the enzyme, its concentration, treatment temperature and time) were studied. In a preliminary study, Bioguavase applied at 30 C produced clarified guava juice with the highest yield. Two experimental trials were conducted. First, guava juice was treated with Bioguavase at three concentrations (400, 600, and 800 ppm) and incubated for 12, 24, and 36 h. In the second trial, shorter treatment times (3, 6, 9, and 12 h) were evaluated using the same enzyme concentrations. Post‐treatment, juice samples were clarified by centrifugation and analyzed for vitamin C content, antioxidant capacity, total soluble phenolics, turbidity, and color. Results indicated that treatment with 600 ppm Bioguavase for 12 h produced the clearest juice in the first trial, with no further improvements in yield observed beyond this duration. In the second trial, a 3‐h treatment at 600 ppm was sufficient to achieve optimal clarification. Enzyme application resulted in a reduction in antioxidant capacity (8%–22%) and an increase in total soluble phenolics (8%–15%). These findings demonstrate that treating guava juice with 600 ppm Bioguavase for 3 h at 30 C effectively improves clarity while minimizing nutrient degradation. This protocol is recommended for industrial‐scale guava juice processing to enhance product quality and processing efficiency.

## Graphical abstract

Treating guava puree with 600 ppm Bioguavase for 3 h at 30 C effectively improves clarity while minimizing nutrient degradation. These conditions are suitable for producing a clarified juice by centrifugation after enzyme treatment of the puree. This protocol is recommended for industrial‐scale guava juice processing to enhance product quality and processing efficiency. Percent yield of clarified juice treated at three different enzyme concentrations and four different reaction times up to 12 h. Error bars represent standard deviation for *n* = 9. Different letters within each reaction time represent significant differences at *α* = 0.5.

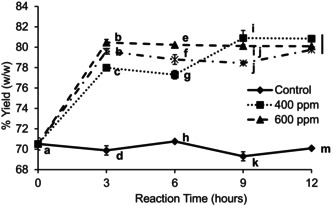

## 1 Introduction

Guava ( *Psidium guajava* , L) is a tropical fruit rich in antioxidants and vitamin C (Youssef and Ibrahim 2016). A member of the Myrtaceae family, it is common to all warm tropical areas of America and can be found in the West Indies, Bahamas, and Bermuda (Nakasone and Paull 1998). Morphologically, the fruit may be round, ovoid, or pear‐shaped with thin, light‐yellow skin, frequently blushed with pink. The flesh can be white, yellowish, light‐ or dark‐pink, or near‐red, juicy, acidic, or sweet and flavorful (Salunkhe and Kadam 1995). Guava can be consumed raw or processed to obtain other products. In Hawaii, it is boiled in slices to produce a juice (Boyle et al. 1957). In Brazil, Mexico, and the Dominican Republic, the fruit is processed to obtain a puree (Rajan and Hudedamani 2019). In South Africa, the fruit is trimmed, minced, and mixed with a natural fungal enzyme to obtain a clear juice without exposure to heat that degrades ascorbic acid and other constituents (Omayio et al. 2019). Guava juice and nectar are among the most popular canned or bottled fruit beverages of the Caribbean area (Morton 1987). Guava puree is generally processed by heat pasteurization to extend its shelf life for up to 1 year, but the fresh taste is modified (Yen and Lin 1996).

The consistency of guava puree has a significant effect on its flow at low temperatures (Vitali and Rao 1982). One way of improving flow is by enzymatic treatment, which reduces the viscosity of the puree. This enhances the degradation and reduction of suspended solids and decreases the viscosity. In the food industry, a combination of enzymes including pectinesterase, arabanase, hemicellulase, tannase, and cellulase is used to degrade the mesocarp of guava, which contains 90% of the total cell wall material as pulp (Kashyap et al. 2001).

Bioguavase (BioSun, Tampa, FL, U.S.A.) is a commercially available enzyme preparation that contains a variety of carbohydrase enzymes derived from *Aspergillus niger*. It causes rapid viscosity reduction of guava puree or fresh guava fruit through pectin hydrolysis, with a significant increase in juice yield. Hydrolysis of pectin produces carboxylic acids and galacturonic acid, which may lead to a pH decrease (Krall and McFeeters 1998). During enzyme treatment, increasing the temperature may produce a well‐clarified juice but may also modify the phytochemical composition and reduce ascorbic acid content due to oxidation.

Our hypothesis was that clarified guava juice could be obtained by enzyme treatment of guava puree under 35°C without significantly affecting its nutritional quality. The objectives of this study were: (1) to evaluate a selection of enzymes with juice clarification properties; (2) to apply a commercial enzyme (Bioguavase) treatment at temperatures below 35°C to obtain a product with a consistency suitable for further processing and an increase in yield, and (3) to optimize (without affecting the phytochemical properties) the time and concentration of Bioguavase for treatment of guava puree in obtaining a clarified product at 30°C.

## 2 Materials and Methods

The experimental design consisted of a preliminary study to apply 5 different enzymes for different reaction times and temperatures. The enzyme with the highest yield (Bioguavase) and a temperature of 30°C was selected. The second part of the study investigated the effects of the Bioguavase enzyme applied for different times on the physicochemical attributes of guava juice.

### 2.1 Preliminary Study

#### 2.1.1 Selection of an Enzyme and a Temperature to Clarify the Guava Puree

Kleryzyme 150 (DSM, Cedex, France), Rapidase TF (ADM, Decatur, IL, U.S.A.), Cellubrix (Novozymes, Denmark), Pectinex Ultra SP‐L (Novozymes, Denmark), Crystalzyme 200XL (Valley Research, South Bend, IN, U.S.A.), Bioguavase (BioSun, Tampa, FL, U.S.A.), Biocranase Super (BioSun, Tampa, FL, U.S.A.) and Biocellulase FG Concentrate (BioSun, Tampa, FL, U.S.A.) were obtained from the manufacturers. Five different enzyme treatments (Table 1) and three different temperatures were used: 12.4°C, 21.4°C, and 30°C. One hundred grams of puree (97% moisture content) were treated with the enzyme preparation at each temperature. Sodium azide (0.002%) was added to each sample. Each sample was incubated at one of the three selected temperatures for a period of 24 ± 1 h. Samples were observed and mixed periodically. After 24 h, the purees were removed from incubation, centrifuged in a Sorvall RC‐5B Refrigerated Superspeed Centrifuge (DuPont Instruments, Newton CT, U.S.A.) at 10,410 ***g*** (9500 rpm) for 8 min at 4°C, and the supernatant was used. The percent yields of juice were calculated using the following equation:

$$\%\text{yield}=\left[\left(\text{initial weight}–\text{weight after centrifugation}\right)/\text{initial weight}\right]\times 100$$

**TABLE 1:** Enzyme and concentration used during enzymatic treatment.

Enzyme treatment code	Enzyme or mixture of enzyme	Concentration of enzyme used
1	Klerzyme 150	300 ppm
	Rapidase TF	600 ppm
3	Crystalzyme 200XL	600 ppm
4	BioGuavase	600 ppm
5	Biocranase Super	600 ppm
	Biocellulase FG Con.	600 ppm

Two replications were performed. The enzyme used for the rest of the study was selected based on the maximum yield of these treatments.

### 2.2 Effects of Bioguavase Treatment at 30°C on Phytochemical Levels in Guava Puree

#### 2.2.1 Sample Preparation

Untreated guava puree obtained from Hawaii (Kai Guava, Kilauea Agronomics, Kilauea, HI, U.S.A.) through a distributor in Florida was transported frozen to the Food Science and Human Nutrition Department in Gainesville, Florida. The puree was thawed, placed into 2 L glass bottles, and immediately frozen at −20°C. Before clarification, the puree was thawed overnight at 6°C. Bioguavase (600 ppm) was used to treat the puree for 24 h at 30°C. Following treatment, the purees were placed in ice slush immediately to stop the enzyme reaction.

#### 2.2.2 Analyses

Analysis of the puree before exposure to temperature (sample before temperature treatment = no‐heat, no‐enzyme (NHNE)) was performed. A control sample of puree exposed to 30°C for the same amount of time without enzyme (heated no‐enzyme, control) was included. The purees were analyzed (described later) for percent juice yield, vitamin C, antioxidant capacity (ORAC), total phenolics, color, and total soluble solids (TSS). Two replications were performed.

### 2.3 Enzyme Optimization to Produce a Clarified Guava Juice

#### 2.3.1 Sample Preparation and Enzyme Treatment

Puree (400 g) was weighed and placed in a beaker. The amount of enzyme added to each beaker was as follows: 160 μL Bioguavase enzyme (400 ppm), 240 μL Bioguavase enzyme (600 ppm), and 320 μL Bioguavase enzyme (800 ppm). These samples, divided into four beakers (each containing 100 g) were placed at 30°C in an incubator. Every 12 h, one beaker was removed from each enzyme concentration and placed in an ice bath to stop the enzyme reaction. These were kept on ice until analyzed. The experiment was performed in duplicate. The previous experiment was conducted again, but sampling time was reduced to every 3 h for up to 12 h. After treatment, all purees were assayed for percent juice yield, vitamin C, antioxidant capacity, total phenolics, turbidity, pH, total soluble solids (TSS, ^o^Brix), and color.

#### 2.3.2 Physicochemical Analyses

##### 2.3.2.1 Percent Juice Yield

Juice yield followed the procedure previously stated.

##### 2.3.2.2 Vitamin C

The 2,6‐dichloroindophenol dye was obtained from Sigma‐Aldrich (St. Louis, MO, U.S.A.), and the acetic and m‐phosphoric acids were obtained from Fisher Scientific (U.S.A.). Vitamin C of the centrifuged samples was assayed by titration using the Official Method published by the AOAC (AOAC method 967.21 1990). Two mL aliquots were used for the titration, and the vitamin C content in each sample was calculated as mg of vitamin C per 100 g sample.

##### 2.3.2.3 Antioxidant Capacity

AAPH (2,2′‐azobis (2‐methylpropionamidine dihydrochloride)), fluorescein (free acid) and Trolox (6‐hydroxy‐2,5,7,8‐tetramethylchroman‐2carboxilic acid) were obtained from Sigma‐Aldrich (St. Louis, MO, U.S.A.). The antioxidant capacity of hydrophilic compounds in the supernatants of centrifuged samples was determined by the oxygen radical absorbance capacity (ORAC) assay (Huang et al. 2005). Antioxidant capacity was calculated by integrating the area under the fluorescence decay curve in the presence of guava phytochemicals and calibrated with a standard curve of Trolox using a SpectraMax Gemini XPS microplate spectrofluorometer (Molecular Devices; Sunnyvale, CA, U.S.A.) and SoftMax Pro 5.2 software (Molecular Devices; Sunnyvale, CA, U.S.A.). Results were expressed as Trolox equivalents (TE) per mL (μmol of TE/mL). A dilution of 100 X was used to obtain the correct area from the treated sample.

##### 2.3.2.4 Total Phenolic Compounds

Total phenolic compounds of the centrifuged samples were analyzed using the Folin–Ciocalteu metal reduction assay (Talcott et al. 2000), using gallic acid as the standard. For analysis, 100 μL of sample was used. Absorbances at 765 nm were taken using a Spectra Max 190 spectrophotometer (Molecular Devices; Sunnyvale, CA, U.S.A.). Gallic acid and Folin–Ciocalteu's reagent were purchased from Sigma‐Aldrich (St. Louis, MO, U.S.A.). All samples were diluted 10 X in order to obtain the absorbance reading within the standard curve.

##### 2.3.2.5 Turbidity (Clarity)

Sample turbidity was measured using the percent transmission mode at 650 nm in a Beckman UV–VIS scanning spectrophotometer (Model # DU‐620, Beckman Coutler; Brea, CA, U.S.A). Clarity of samples was determined using 1.5 mL of sample in 1 cm plastic disposable cuvettes against water.

##### 2.3.2.6 pH

The pH was measured using an Orion expandable ion analyzer EA 920 pH meter (Orion Research; Boston, MA, U.S.A.) equipped with an Accumate glass electrode (Fisher Scientific; U.S.A.) Samples (10 mL) were placed in a 50 mL beaker. The beaker was placed over a stirring plate, and the samples were stirred during the measurements. The electrode was immersed in the puree, and a reading was taken once the pH meter reading was steady.

##### 2.3.2.7 Total Soluble Solids (TSS)

^o^Brix of the centrifuged juice was measured using an electronic ABBE Mark II refractometer at room temperature (Leica Inc.; Buffalo, NY, U.S.A.).

##### 2.3.2.8 Color Analysis

Color of the centrifuged juice was measured using a Gardner colorimeter (BYK‐Gardner USA; Columbia, MD, U.S.A.) and expressed as L*, a*, and b*. Sample (20 mL) used to perform the analysis was measured in the reflectance mode.

### 2.4 Statistical Analysis

SAS 9.0 software (SAS Institute Inc.; Cary, NC, U.S.A.) was utilized for all statistical analyses. For the selection of an enzyme treatment and temperature, analysis of variance (ANOVA) for factorial design was employed. To study the effects of enzyme treatment at low temperature on phytochemical levels in guava puree, one‐way ANOVA was employed with mean separation by Tukey's standardized test. For both experiments conducted on the enzyme optimization treatment for guava puree, a repeated measurement design was used. Chemical measurement data for the treated and untreated samples were analyzed by a Tukey's standardized range (HSD) test at a significant level of *α* = 0.05.

## 3 Results and Discussion

### 3.1 Preliminary Experiments: Selection of Enzyme and Temperature

**FIGURE 1:**
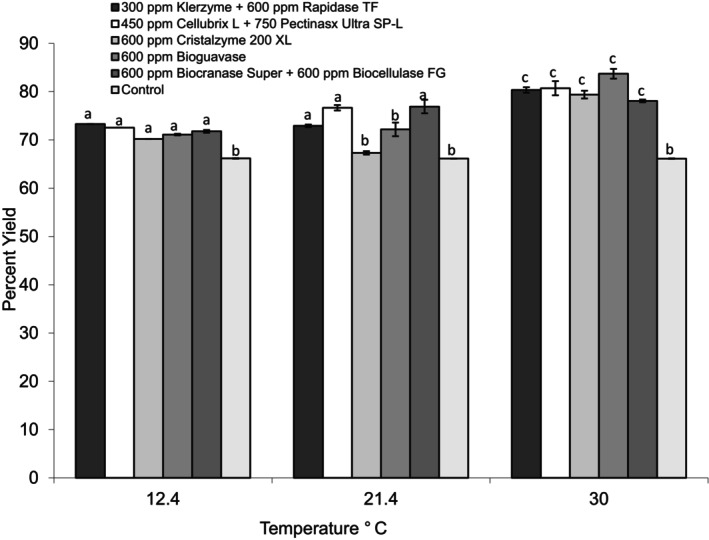
Percent yield for each enzyme treatment at three different temperatures (Treatment time: 24 h). Error bars represent *n* = 4. Different letters within each reaction temperature represent significant differences at *α* = 0.5.

A control sample of puree without enzyme was subjected to the same incubation temperature and centrifugation step as treatments. The yield for the control puree was 66.14% ± 0.03%, irrespective of the incubation temperature. The enzyme treatment with the highest yield (83.72% ± 1.02%) was treatment 5, or 600 ppm Bioguavase (Figure 1). All enzyme treatments resulted in a higher yield at 30°C (86°F) compared to the control. There was no significant difference (*α* = 0.05) between the type of enzyme and the percent yield, but there was a significant difference (*α* = 0.05) between the temperature and percent yield. All enzymes have a temperature range where they work under optimum conditions. All enzymes used during the first part of this research were active at temperatures between 10°C and 55°C. Enzyme activity increased with temperature to a certain point, and then inactivation occurred. The selection of the temperature and enzyme (Bioguavase) for further experiments was based solely on the higher yield it produced.

### 3.2 Effect of Enzyme Treatment on Chemical Composition of a Clarified Guava Juice

The yield for the non‐heated, no‐enzyme (NHNE) puree was 72.18%. The heated no‐enzyme or control (30°C for 24 h) showed a slightly increased yield of 72.92%, and the enzyme‐treated (ET) puree (heated with enzyme) had a yield of 82.93%. Thus, the increase in the yield of the enzyme‐treated sample compared to controls was not due to the treatment temperature (Table 2). There were significant differences (*α* = 0.05) between the NHNE puree and ET puree and between the control and ET puree (based on ANOVA analysis). As expected, the use of enzymes to clarify juices increases the yield of the final product. A key component of commercial pectinases is pectin methyl esterase, converting colloidal pectin to noncolloidal pectic acid. This results in the sedimentation of cloud‐forming particles. In apple juice production, pectinases are added with the objective of producing a high‐yield clear juice (Christen and Smith 2000). In sparkling clear juice production, enzymes are added to increase the juice yield during pressing and straining of the juice and to remove suspended matter (Kashyap et al. 2001).

**TABLE 2:** Percent yield, ORAC value, total soluble phenolics, and ascorbic acid content of guava puree (600 ppm of Bioguavase, 30°C and 24 h of reaction time).

Sample	% Yield	ORAC (μmol TE/mL)	Total soluble phenolics (GAE)	Ascorbic acid (mg AA/100 g)
Non‐heated, no‐ enzyme (NHNE)	72.78 ± 0.27^a^	13.27 ± 1.56^a^	837.46 ± 37.0256^a^	81.57 ± 0.0056^a^
Heated no‐enzyme (control)	72.92 ± 0.33^a^	13.78 ± 3.62^a^	910.21 ± 59.5756^b^	80.72 ± 0.8656^a^
Heated + enzyme (ET)	82.93 ± 0.32^b^	12.63 ± 0.98^a^	892.96 ± 56.9456^b^	79.81 ± 2.2456^a^

*Note:* Same letters within the column means no significant differences.

Table 2 presents the results for the antioxidant capacity measurements. Before the temperature treatment, the antioxidant capacity was 13.27 μmol TE/mL. The antioxidant capacity for the control and the ET purees was 13.78 and 12.63 μmol TE/mL, respectively. There were no significant differences (*α* = 0.05) between the three samples, indicating that neither the enzyme nor temperature influenced the antioxidant capacity of the product. These results are comparable to a previous study (Fender 2005) where 10.5 μmol TE/mL was obtained in guava nectar.

Total soluble phenolics were measured by the Folin–Ciocalteu assay, which measures the capacity of phytochemical compounds to reduce an oxidized metal ion. Although the target compounds for this assay were polyphenolics, compounds such as ascorbic acid, certain soluble proteins, melanoidin, and reducing sugars can interfere in the assay. The average total soluble phenolics, expressed in Gallic Acid Equivalent (GAE), is presented in Table 2. Total soluble phenolics in the NHNE sample were 837.46 mg/L GAE. The control and the ET samples had total soluble phenolics content of 910.21 and 892.96 mg/L GAE, respectively. There was an increase in total phenolic content due to temperature treatment and enzyme activity. Comparing the control to the enzyme‐treated puree, there was a small decrease in total phenolic content in the enzyme‐treated sample. There were significant differences (*α* = 0.05) between the NHNE and the control, which showed no significant difference to the ET sample.

Guava has one of the highest concentrations of vitamin C among all fruits, typically containing between 200 and 300 mg vitamin C/100 g. In a study conducted on guava juice processing optimization (Chopda and Barrett 2001), the authors found an ascorbic acid content of 149.4 mg ascorbic acid/100 g sample in the supernatant. Table 2 presents the mg of ascorbic acid/100 g sample before (NHNE) and after temperature treatment (enzyme and no enzyme). The ascorbic acid content for the NHNE sample was 81.57 mg ascorbic acid/100 g sample (Table 2). The ascorbic acid content decreased due to the temperature effect, probably due to oxidation reactions. The control shows an ascorbic acid content of 80.72 mg, and the ET puree had an ascorbic acid content of 79.81 mg/100 g. Although ascorbic acid content decreased with increasing temperature, there were no significant differences (*α* = 0.05) between the three samples. This indicated that the conditions of enzyme processing did not create an environment leading to ascorbic acid oxidation or degradation. The ascorbic acid content was lower than that reported in other literature. In this study, the puree did not contain the guava skin, which is the part of the fruit with the highest ascorbic acid content.

Table 3 shows the results of color analysis. The L* values (lightness) for heated‐no enzyme (49.66), NHNE (48.73), and ET (48.23) are statistically different (*α* = 0.05). However, the decrease in the L* value for the clarified juice samples is very large. After centrifugation, the NHNE value reduced to 32.83, that of the control to 29.56, and ET to 11.33. The redness (a*) of the samples before centrifugation was high and close to each other (NHNE = 22.44, Control = 22.84, ET = 22.35). Enzyme treatment of guava puree causes the liberation of compounds such as carotenoids, which are bound to complex carbohydrates (components of the plant cell wall) and broken down during clarification by the enzyme (Steven 1998). There were significant differences (*α* = 0.05) in redness between the three samples. Again, the decrease in the a* values after centrifugation was very large (NHNE = −0.98, control = −1.68, ET = −0.77). The yellowness (b*) of the samples before centrifugation was close (NHNE = 14.73, control = 14.79, ET = 14.50). However, the decrease of b* after centrifugation was very large (NHNE = −5.61, control = −5.98, ET = −1.95).

**TABLE 3:** Color values obtained for three different guava purees before and after clarification (600 ppm of Bioguavase, 30°C and 24 h of reaction time).

Sample	L* value		a* value		b* value	
	Puree	Clarified puree	Puree	Clarified puree	Puree	Clarified puree
Non‐heated, no‐enzyme (NHNE)	48.73 ± 0.01^a^	32.83 ± 0.32^a^	22.44 ± 0.03^a^	−0.98 ± 0.07^a^	14.73 ± 0.01^a^	−5.61 ± 0.20^a^
Heated no‐enzyme (control)	49.66 ± 0.01^b^	29.56 ± 0.68^b^	22.84 ± 0.15^b^	−1.68 ± 0.15^b^	14.97 ± 0.31^b^	−5.98 ± 0.19^b^
Heated + Enzyme (ET)	48.23 ± 0.29^c^	11.33 ± 0.93^c^	23.25 ± 0.25^c^	−0.77 ± 0.23^c^	14.50 ± 0.19^c^	−1.95 ± 0.30^c^

*Note:* Same letters within the column means no significant differences.

The ΔE value quantifies color differences between two samples (Schuessler 2020). ΔE < 1 indicates color difference is imperceptible to the human eye. ΔE > 3 signifies a human‐noticeable difference. ΔE between NHNE and control was 1.04, ΔE between control and ET was 1.56, and ΔE between NHNE and ET was 0.98. This implies the closeness of colors pre‐centrifugation.

The centrifugation process reduced the L*, a*, and b* for all three samples, but the redness and yellowness of the samples were the most affected. Centrifugation removed most of the insoluble particles, which could have contained the compounds responsible for the pink color, but some colloidal particles remained, causing turbidity in the non‐heated sample and the control juice. The enzyme‐treated puree produced a clear juice after the centrifugation process. Centrifugation of the NHNE and control samples produced a cloudy supernatant, while the ET puree produced a clear juice (supernatant). Post‐centrifugation, ΔE between NHNE and control was 3.36, ΔE between control and ET was 18.69, and ΔE between NHNE and ET was 21.81.

Centrifugation decreased the total soluble solids content (^o^Brix) of the samples (Table 4). Before temperature treatment, NHNE had a°Brix of 7.1, while the control and enzyme‐treated purees had 7.2° and 7.4°Brix, respectively. After centrifugation, the NHNE sample was 5.7°Brix, while the temperature‐treated control and enzyme‐treated samples were 6.5° and 6.6°Brix, respectively. There were significant differences (*α* = 0.05) between the°Brix for the purees and the clarified samples. As expected, °Brix increases due to temperature and enzyme treatment. The increase in°Brix is due to the breakdown of pectin and other complex carbohydrates. In the case of pectin breakdown, a release of galacturonic acid (pectic acid) occurs, which is soluble and contributes to the increase in the°Brix measurement. Pectic substances or pectin are high molecular weight polysaccharides found in plant cell wall middle lamellae. They are composed of galacturonic acid units, joined by α‐1,4 glycosidic linkages. Some of the acid groups, along with the acid units, become methylated during the fruit ripening (Murano 2003). When pectinases are added to fruit juices, the conversion of insoluble pectin to soluble pectic acid occurs (Christen and Smith 2000).

**TABLE 4:** Total soluble solids for guava puree before and after clarification for the three different treatments (600 ppm of Bioguavase, 30°C and 24 h of reaction time).

Sample	Brix	
	Puree	Clarified
Non‐heated, no‐ enzyme (NHNE)	7.1 ± 0.056^a^	5.7 ± 0.0^b^
Heated, no‐enzyme (control)	7.2 ± 0.056^a^	6.5 ± 0.0^c^
Heated + Enzyme (ET)	7.4 ± 0.056^a^	6.6 ± 0.0^c^

*Note:* Same letters within the column means no significant differences.

### 3.3 Enzyme Treatment Optimization to Produce a Clarified Guava Juice

Results for the physicochemical analyses conducted on guava puree after treatment with 400, 600, and 800 ppm of Bioguavase enzyme over three reaction times (12, 24, and 36 h) are presented in Table 5. There were significant differences due to the reaction time, enzyme concentration, and their interaction (time × concentration). Regarding yield, there was a significant difference between the control (zero enzyme concentration and reaction time, 61.27%) and the enzyme treatments (up to 67.8% for no enzyme at 36 h). There were no significant differences between the three enzyme concentrations for every reaction time. Also, after 12 h, the increase in yield was not significant for any of the three enzyme concentrations. Increasing enzyme concentration will increase the rate of the reaction. After 12 h of reaction time, the maximum reaction rate was achieved, and there were no further differences between the three enzyme concentrations. To reduce the reaction time and obtain maximum juice yield, another study was performed. In this study, samples were taken every 3 h for up to 12 h.

**TABLE 5:** Physicochemical results for enzymatic treatment of guava puree at three different concentrations and three different reaction times (Reaction temperature: 30°C).

Physicochemical analysis	Enzyme concentration (ppm)	Reaction time (hours)			
		0	12	24	36
% Yield	0	61.27 ± 0.14^a^	60.44 ± 0.26^a^	65.72 ± 0.28^a^	67.80 ± 0.40^a^
	400	61.27 ± 0.14^a^	76.44 ± 1.44^b^	77.43 ± 1.61^b^	77.55 ± 1.95^b^
	600	61.27 ± 0.14^a^	79.21 ± 0.82^b^	79.99 ± 0.84^b^	80.19 ± 1.19^b^
	800	61.27 ± 0.14^a^	79.30 ± 1.32^b^	80.58 ± 0.46^b^	79.49 ± 0.24^b^
Vitamin C (mg ascorbic acid/100 g sample)	0	71.80 ± 0.00^a^	71.12 ± 0.01^a^	68.52 ± 0.00^a^	66.01 ± 0.02^a^
	400	71.80 ± 0.00^a^	75.56 ± 0.01^b^	69.20 ± 0.00^a^	66.50 ± 0.01^a^
	600	71.80 ± 0.00^a^	74.98 ± 0.01^b^	68.90 ± 0.00^a^	66.01 ± 0.02^a^
	800	71.80 ± 0.00^a^	74.98 ± 0.00^b^	70.26 ± 0.00^a^	66.21 ± 0.01^a^
Antioxidant Capacity (μMol TE/L)	0	11.86 ± 0.92^a^	13.30 ± 0.69^a^	12.36 ± 0.27^a^	11.79 ± 1.88^a^
	400	11.86 ± 0.92^a^	10.91 ± 0.32^a^	11.50 ± 0.61^ab^	10.03 ± 0.75^a^
	600	11.86 ± 0.92^a^	10.28 ± 0.82^b^	10.98 ± 1.03^b^	9.61 ± 1.70^a^
	800	11.86 ± 0.92^a^	8.79 ± 0.31^c^	9.01 ± 1.15^c^	10.06 ± 1.79^a^
Total Soluble Phenolics Compounds (GAE)	0	889.10 ± 6.80^a^	786.81 ± 28.63^a^	757.50 ± 18.45^a^	834.90 ± 18.45^a^
	400	889.10 ± 6.80^a^	878.61 ± 39.38^b^	869.86 ± 25.83^b^	913.44 ± 25.83^b^
	600	889.10 ± 6.80^a^	874.03 ± 54.63^b^	848.89 ± 21.51^b^	962.71 ± 21.51^b^
	800	889.10 ± 6.80^a^	825.28 ± 61.83^ab^	823.19 ± 59.53^b^	926.67 ± 59.53^b^
Turbidity	0	23.33 ± 0.00^a^	25.68 ± 1.57^a^	24.42 ± 0.39^a^	29.36 ± 0.41^a^
	400	23.33 ± 0.00^a^	72.04 ± 3.00^b^	70.16 ± 5.37^b^	80.95 ± 1.07^b^
	600	23.33 ± 0.00^a^	86.29 ± 3.47^c^	80.34 ± 6.52^c^	82.48 ± 2.29^b^
	800	23.33 ± 0.00^a^	76.39 ± 4.66^b^	69.59 ± 2.84^b^	85.14 ± 4.56^b^
pH	0	3.10 ± 0.00^a^	3.17 ± 0.16^a^	3.15 ± 0.01^a^	3.15 ± 0.01^a^
	400	3.10 ± 0.00^a^	3.11 ± 0.00^b^	3.09 ± 0.01^b^	3.09 ± 0.02^b^
	600	3.10 ± 0.00^a^	3.08 ± 0.01^c^	3.07 ± 0.00^c^	3.08 ± 0.01^c^
	800	3.10 ± 0.00^a^	3.05 ± 0.01^d^	3.01 ± 0.01^d^	3.05 ± 0.01^d^
Titratable Acidity (mg of citric acid/100 g)	0	0.91 ± 0.01^a^	0.92 ± 0.01^a^	0.93 ± 0.02^a^	0.92 ± 0.02^a^
	400	0.91 ± 0.01^a^	0.98 ± 0.01^b^	0.97 ± 0.02^b^	0.97 ± 0.01^b^
	600	0.91 ± 0.01^a^	0.97 ± 0.02^b^	0.97 ± 0.01^b^	0.98 ± 0.01^b^
	800	0.91 ± 0.01^a^	0.96 ± 0.01^b^	0.97 ± 0.01^b^	0.98 ± 0.01^b^
Total Soluble Solids (Brix)	0	6.40 ± 0.00^a^	6.40 ± 0.00^a^	6.40 ± 0.00^a^	6.40 ± 0.00^a^
	400	6.40 ± 0.00^a^	6.70 ± 0.30^b^	6.70 ± 0.20^b^	6.70 ± 0.10^b^
	600	6.40 ± 0.00^a^	6.70 ± 0.10^b^	6.70 ± 0.00^b^	6.80 ± 0.10^c^
	800	6.40 ± 0.00^a^	6.60 ± 0.00^ab^	6.70 ± 0.00^b^	6.70 ± 0.00b^c^

*Note:* Same letters within the column means no significant differences.

#### 3.3.1 Vitamin C

In Table 5, there were significant differences in vitamin C content due to the reaction time and the interaction of time with enzyme concentration. At 12 h, the control had lower vitamin C when compared to all levels of enzyme (control = 71.12, 800 ppm =74.98 mg/100 g). At 24 and 36 h this difference decreased (control = 68.52, 800 ppm =70.26 mg/100 g; control = 66.01, 800 ppm = 66.21, respectively). Comparing the ascorbic acid content reported in the literature, the values of this study were lower. Vitamin C content depends on the variety of guava, harvest season, and processing conditions. Vitamin C in the fresh fruit is always higher than in the puree.

#### 3.3.2 Antioxidants

Antioxidants are important in foods for their potential health benefits and their radical scavenging abilities. They can prevent oxidation in foods and in the human body. There were significant differences in antioxidant activity due to the enzyme concentration, reaction time, and the interaction (Table 5). Antioxidant capacities for the control (no enzyme) were higher compared to the other enzyme concentrations. At 12 h, the antioxidant capacity decreased with the amount of enzyme used (no enzyme = 13.3, 800 ppm = 8.79 μMol TE/L), but there were no significant differences in the ORAC value between 400 and 600 ppm. At 24 h, there were no significant differences between the control, 400, and 600 ppm enzyme concentrations. At 36 h, there was no significant difference between all levels of enzyme concentration.

#### 3.3.3 Total Phenolics

Results of the Folin–Ciocalteu assay for total phenolics revealed that there were significant differences due to the reaction time, enzyme concentration, and their interaction. At 12 h, the total phenolics in the control (786.81) were not significantly different from the 800 ppm enzyme (825.28 GAE), and there were no significant differences between the three levels of enzyme concentrations. At 24 and 36 h, there were significant differences between the control and the three enzyme concentrations (400, 600, and 800 ppm). There was an increase in total phenolics content due to the enzyme concentration, and a larger amount of total soluble phenolics was observed at 36 h reaction time. The increase in total phenolics is due to the release, by enzymatic reaction, of phenolic compounds from the pulp. Jiménez‐Escrig et al. (2001) tested the fiber from the pulp and peel of guava from Caracas. They found that both fibers were potent sources of radical‐scavenging compounds, presumably from the high content of cell‐wall‐bound polyphenolics reported for each fiber. They found pulp fiber had a total extractable phenol content of 26.2 g GAE/kg dry matter.

#### 3.3.4 Turbidity

Turbidity is a key factor in clarified juice. The results showed that the use of enzyme is adequate to obtain a clarified juice (Table 5). There were significant differences regarding turbidity based on the reaction time, enzyme concentration, and their interaction. The control had the lowest percent transmittance (lower clarity). The juice was cloudy at time 0 (23.33) and showed an increase in turbidity at 36 h (29.36). The 400, 600, and 800 ppm enzyme treatments at 12, 24, and 36 h reaction times had turbidity values higher than at 0 h. At 12 and 24 h, there were no significant differences in turbidity between 400 and 800 ppm samples. At 36 h, there were no differences between 400, 600, and 800 ppm samples. As expected, enzyme treatment produced a clear juice due to the conversion of colloidal pectin to noncolloidal pectic acid. Colloidal pectin is responsible for the cloudy appearance of fruit juices, and production of acid results in the sedimentation of the cloud‐forming particles (Christen and Smith 2000).

#### 3.3.5 pH

There were significant differences in pH due to the enzyme concentration, reaction time, and their interaction (Table 5). During the first 12 h of reaction, the pH decreased with increasing enzyme concentration (3.11 at 400 ppm, 3.05 at 800 ppm). After 24 h, the pH remained almost constant with reaction time, regardless of enzyme concentration. Enzyme treatment decreased the pH of the product due to the release of galacturonic acid.

#### 3.3.6 °Brix

The°Brix of the clarified guava juice was between 6.4 and 6.7. There were significant differences due to enzyme concentration, reaction time, and their interaction (Table 5). TSS for the control stayed the same over time (6.4), but the°Brix increased with enzyme concentration during treatment. An enzyme concentration of 400 ppm did not cause a further increase in°Brix from 6.7 after 12 h reaction time. However, 600 ppm of enzyme concentration caused a further increase in°Brix at 36 h (6.8), this value being the highest. The increase in°Brix was related to a decrease in pH and a decrease in turbidity since all are related to pectin breakdown.

#### 3.3.7 Color

Color analysis results are summarized in Table 6. At 400 ppm enzyme concentration, L* values decreased with treatment times of 12, 24, and 36 h; from 27.29 at 12 h to 26.61 at 24 h, to 23.17 at 36 h. The a* value increased after the clarification process during the first 12 h, but then decreased. There were no differences between the three levels of enzyme concentration during the first 24 h of reaction time. The b* values increased throughout the clarification process at 400, 600, and 800 ppm. At 400 ppm the b* value increased from −3.46 at time 0 to 2.12 at 36 h. At 600 ppm the increase was from 3.46 at time 0 to 1.9 at 36 h. At 800 ppm, the increase was from 3.46 to 1.98.

**TABLE 6:** Color results for enzymatic treatment of guava puree at three different concentrations and three different reaction times.

	Enzyme concentration (ppm)	Reaction time (hours)			
		0	12	24	36
L* values	0 ppm	38.77 ± 0.0^a^	42.36 ± 0.96^a^	43.32 ± 0.67^a^	37.23 ± 0.13^a^
	400 ppm	38.77 ± 0.0^a^	27.29 ± 1.01^b^	26.61 ± 0.49^b^	23.17 ± 0.78^b^
	600 ppm	38.77 ± 0.0^a^	24.43 ± 0.81^c^	24.86 ± 1.12^c^	20.67 ± 0.12^c^
	800 ppm	38.77 ± 0.0^a^	26.61 ± 0.5^b^	27.66 ± 0.35^b^	22.69 ± 0.31^b^
a* values	0 ppm	−2.63 ± 0.04^a^	−1.75 ± 0.10^a^	−2.29 ± 0.10^a^	−2.92 ± 0.07^a^
	400 ppm	−2.63 ± 0.04^a^	−1.58 ± 0.09^a^	−2.00 ± 0.05^b^	−2.34 ± 0.21^b^
	600 ppm	−2.63 ± 0.04^a^	−1.91 ± 0.10^b^	−2.01 ± 0.05^b^	−2.37 ± 0.07^b^
	800 ppm	−2.63 ± 0.04^a^	−1.84 ± 0.07^b^	−2.02 ± 0.09^b^	−2.04 ± 0.16^c^
b* values	0 ppm	−3.46 ± 0.02^a^	−4.64 ± 0.24^a^	−3.69 ± 0.35^a^	−3.18 ± 0.03^a^
	400 ppm	−3.46 ± 0.02^a^	−0.90 ± 0.303^b^	0.54 ± 0.25^b^	2.12 ± 0.60^b^
	600 ppm	−3.46 ± 0.02^a^	1.05 ± 0.48^c^	1.38 ± 0.45^c^	1.9 ± 0.07^b^
	800 ppm	−3.46 ± 0.02^a^	−0.13 ± 0.04^d^	−0.162 ± 0.1^d^	1.98 ± 0.41^b^

*Note:* Same letters within the column means no significant differences.

### 3.4 Additional Study on Clarified Guava Puree for Reaction Times of 3, 6, 9, and 12 h

#### 3.4.1 Yield

**FIGURE 2:**
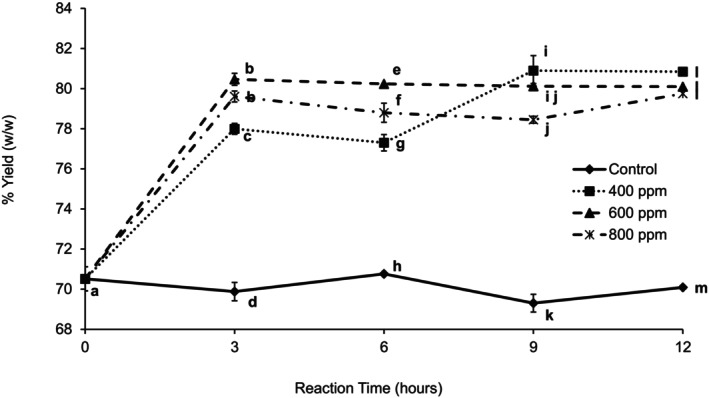
Percent yield of clarified juice treated at three different enzyme concentrations and four different reaction times up to 12 h. Error bars represent standard deviation for *n* = 9. Different letters within each reaction time represent significant differences at *α* = 0.05.

The food industry uses enzymes to increase juice yield. Figure 2 shows the yield (%) obtained for the control and each enzyme concentration (400, 600, and 800 ppm) over reduced reaction times (3, 6, 9, and 12 h). Enzyme treatment significantly increased juice yield. Four hundred ppm slowly increased juice yield, but after 9 h there were no significant differences between 400 and 600 ppm samples. Imungi et al. (1980) clarified guava puree using 400 ppm of Pectinex superconcentrate at a temperature between 40°C and 50°C. They found that the yield increased with treatment time, and 90 min was adequate to achieve maximum yield without decreasing vitamin C content (Imungi et al. 1980). Sandhu and Bhatia (1985) also observed an increase in juice yield with enzyme treatment due to a considerable reduction of pectin. Brasil et al. (1995) observed an increase in juice yield with increasing treatment time when treating guava puree with 600 ppm Clarex‐L super‐concentrate at 45°C for up to 150 min.

**FIGURE 3:**
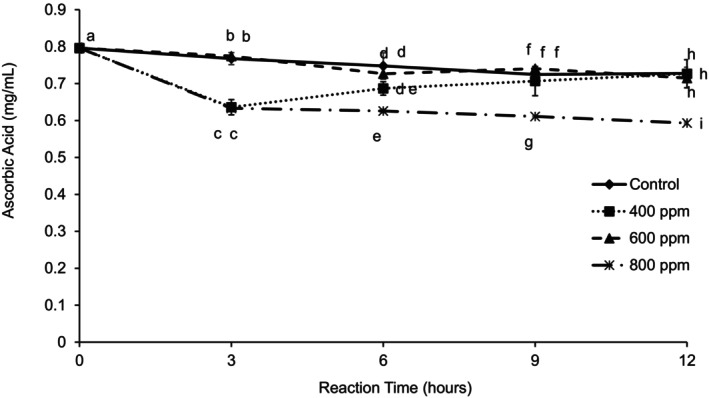
Ascorbic acid content of clarified guava juices after guava puree was treated at three different enzyme concentrations and four different reaction times up to 12 h. Error bars represent standard deviation for *n* = 9. Different letters within each reaction time represent significant differences at *α* = 0.05.

There were no significant differences in the vitamin C content between the control and the puree clarified with 600 ppm enzyme concentration (Figure 3). Enzyme concentration (400 and 800 ppm) significantly decreased the vitamin C content during the first 3 h reaction time. After 3 h, there was an increase in vitamin C content for 400 ppm enzyme concentration, and after 6 h reaction time, there were no significant differences between the vitamin C content for the control, 400, and 600 ppm samples. An enzyme concentration of 800 ppm decreased the amount of vitamin C in the clarified juice probably because the rate of ascorbic acid degradation was greater than its liberation from the pulp. Opposite results were found by Brasil et al. (1995) after treating guava puree with pectic enzyme to obtain a cloudy juice. The reason for this difference may be explained by the type of puree used for their research. They prepared the puree by mashing the fruits using a fruit mill. The enzyme treatment increased the vitamin C content of the cloudy juice due to its liberation, especially from the peel of the fruit which is known to have more vitamin C than the flesh of the fruit (Brasil et al. 1995).

**FIGURE 4:**
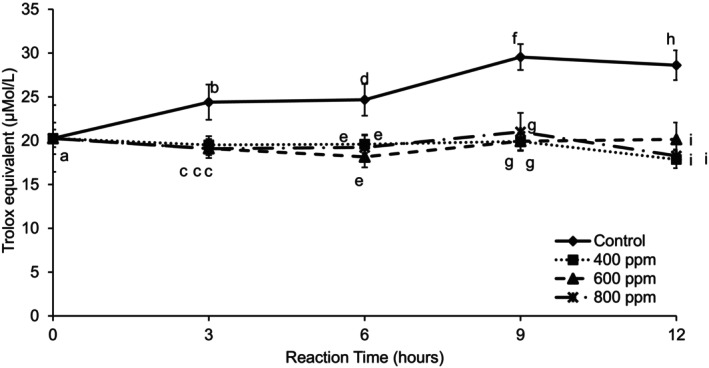
Antioxidant capacity (μMol TE/L) of clarified guava juice treated with three different enzyme concentrations during 12 h of reaction time at 30°C. Error bars represent standard deviation for *n* = 9. Different letters within each reaction time represent significant differences at *α* = 0.05.

The results of the antioxidant capacity analysis are shown in Figure 4. Antioxidant capacity for the control was high compared to enzyme treatments. The antioxidant capacity decreased slightly over time for added enzyme concentrations. The use of enzyme treatment to produce a clarified juice decreased the antioxidant capacity of the guava product. Compounds that have antioxidant capacity are bound to complex carbohydrates and removed after the enzyme treatment followed by centrifugation. Reduction of antioxidant capacity is related to a decrease in vitamin C (which has antioxidant capacity).

**FIGURE 5:**
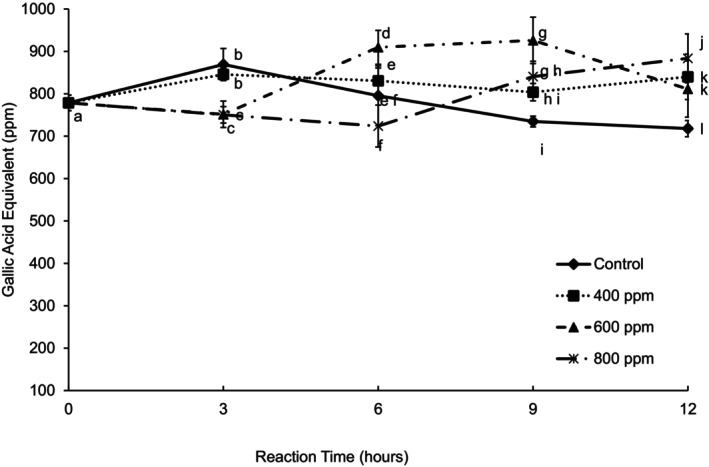
Total soluble phenolics (GAE) of guava juice treated with three different enzyme concentrations during 12 h of reaction time at 30°C. Error bars represent standard deviation for *n* = 9. Different letters within each reaction time represent significant differences at *α* = 0.05.

Figure 5 shows the average total soluble phenolic compounds expressed in Gallic Acid Equivalent (GAE). There were significant differences due to the reaction time, enzyme concentration, and their interaction. At 3 h, the total phenolic levels in the control were not significantly different from the 400 ppm, and the amount of total phenolic compounds at 600 and 800 ppm was lower compared to the control. The total phenolics content decreased over time after a 3 h reaction time. An enzyme concentration of 400 ppm increased total phenolics during the first 3 h reaction time. There was a slight reduction in total phenolics content up to 6 h reaction time, and after 6 h, there was no significant change. There was a significant increase in total phenolics after a 3 h reaction time for 600 ppm enzyme concentration. The content stayed unchanged up to 9 h reaction time, after which the content significantly dropped to a level similar to the enzymatic treatment of 400 ppm. Imungi et al. (1980) found an increase in total phenolics after enzyme treatment of guava puree, but with the clarification process (filtration) used during the research, a significant decrease in this content was observed.

**FIGURE 6:**
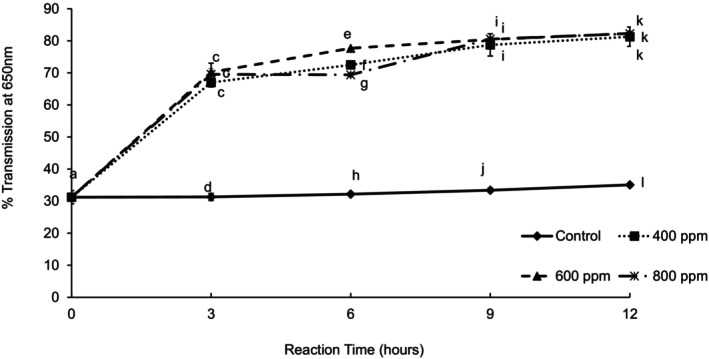
Turbidity (% transmission at 650 nm) of guava juice treated with three different enzyme concentrations during 12 h of reaction time at 30°C. Error bars represent standard deviation for *n* = 9. Different letters within each reaction time represent significant differences at *α* = 0.05.

The results obtained from the turbidity analysis (Figure 6) showed that the use of enzyme resulted in a clarified juice. There were significant differences between the reaction time, enzyme concentration, and their interaction. The control had the lowest percent transmittance, and the juice remained cloudy.

**FIGURE 7:**
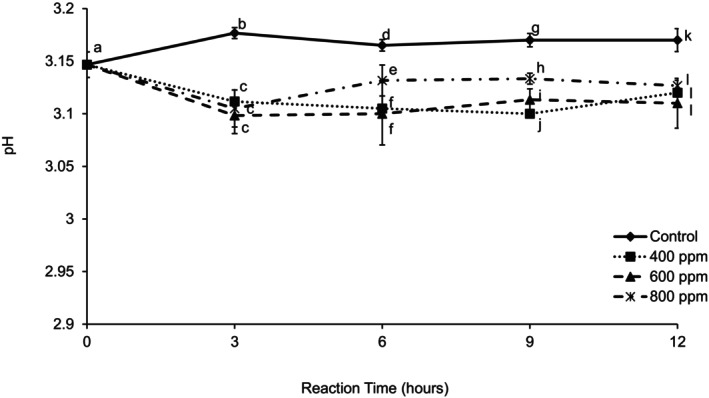
pH of clarified guava juice treated with three different enzyme concentrations during 12 h of reaction time at 30°C. Error bars represent standard deviation for *n* = 9. Different letters within each reaction time represent significant differences at *α* = 0.05.

As seen from the results presented in Figure 7, the pH decreased with enzyme concentration. After 3 h, the pH stayed almost constant regardless of enzyme concentration. The control showed an increase (less acid) in pH during the first 3 h, and after this, the pH was constant. The enzyme treatment decreased the pH of the juice due to the release of galacturonic acid from pectin hydrolysis. A decrease in pH with enzyme treatment of guava puree was reported (Imungi et al. 1980). Chopda and Barrett (2001) observed a decrease in pH as enzyme concentration and incubation time increased at an incubation temperature of 50°C.

**FIGURE 8:**
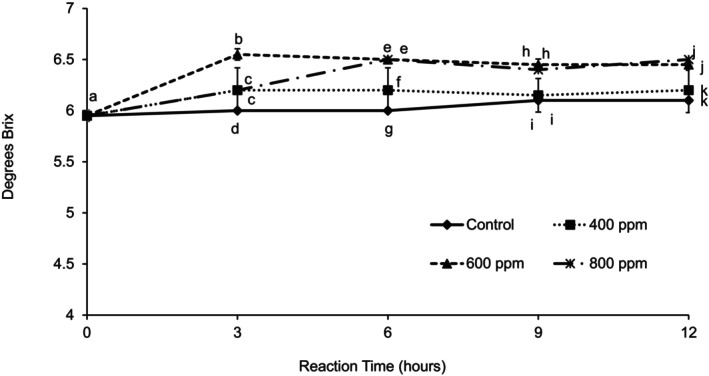
Total soluble solids (°Brix) of clarified guava juice treated with three different enzyme concentrations during 12 h of reaction time at 30°C. Error bars represent standard deviation for *n* = 9. Different letters within each reaction time represent significant differences at *α* = 0.05.

Total soluble solids (Figure 8) showed that the control stayed the same over time, but the°Brix increased with enzyme concentration. An enzyme concentration of 600 ppm did not cause a further increase in°Brix after 3 h reaction time, producing the clarified juice with the highest soluble solid content. After 6 h, there were no significant differences in°Brix between 600 and 800 ppm. After 9 h reaction time, there were no significant differences in total soluble solids between the control and 400 ppm enzyme concentration. The reason for the increase in TSS may be explained by the release of acid during pectin breakdown. An increase in TSS was observed by Thakur and Das Gupta (2006) after extracting beetroot juice from beetroots using Pectinex Ultra SPL 0.15% (1500 ppm) at 45°C for 2.5 h. Chopda and Barrett (2001) observed an increase in°Brix for guava juice as enzyme concentration and incubation time increased at an incubation temperature of 50°C.

**FIGURE 9:**
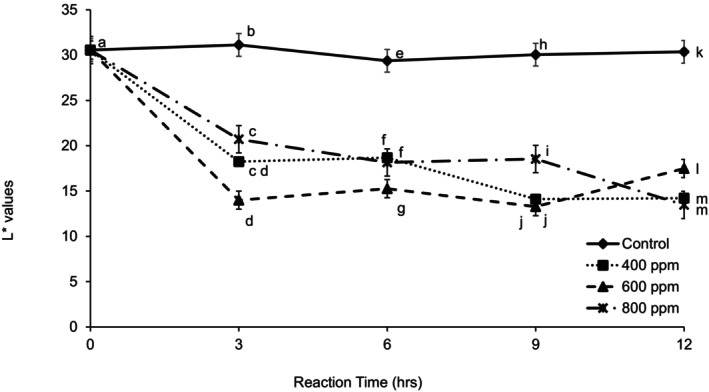
L* values of clarified guava juice treated with three different enzyme concentrations during 12 h of reaction time at 30°C. Error bars represent standard deviation for *n* = 9. Different letters within each reaction time represent significant differences at *α* = 0.5.

As observed in Figure 9, L* values decreased with enzyme concentration, and there were no significant differences between the control and the 3 levels of enzyme concentration at the initial reaction time (0 h). There were no differences between 400 and 600 ppm at 3 and 9 h reaction time. Enzyme treatments reduced the L* value of the samples. The same results were observed by Hodgson et al. (1990) after treating guava puree with Pectinex Ultra Sp‐L at a concentration of 0.2% (2000 ppm) for 2 h at 50°C or 16 h at 20°C. In addition, a decrease in a* value and a slight increase in b* was observed (Hodgson et al. 1990).

## 4 Conclusions

Based on these results, 3 h reaction time and 600 ppm of enzyme concentration are adequate to produce a clarified juice without minimally affecting its nutritional value. These conditions are suitable for producing a clarified juice by centrifugation after enzyme treatment of the puree. The limitation of this procedure resides in using a filtering step to obtain a juice instead of centrifugation. After filtering the enzyme‐treated puree, the color of the juice was much lighter than the original puree. Guava press cake retained all the pink color and may be used to increase the mouthfeel in formulated juices. Other uses for the guava cake, such as a possible source of fiber and natural colorant, should be studied.

## Author Contributions

**M. L. Plaza:** formal analysis (lead), investigation (lead), methodology (lead), writing – original draft (lead), writing – review and editing (supporting). **M. M. Ramírez‐Rodrigues:** writing – review and editing (supporting). **M. O. Balaban:** writing – review and editing (supporting). **M. R. Marshall:** writing – review and editing (supporting).

## Conflicts of Interest

The authors declare no conflicts of interest.

## Data Availability

The data that support the findings of this study are available from the corresponding author upon reasonable request.
